# Influence of Different Segmentations on the Diagnostic Performance of Pericoronary Adipose Tissue

**DOI:** 10.3389/fcvm.2022.773524

**Published:** 2022-03-03

**Authors:** Didi Wen, Rui An, Shushen Lin, Wangwei Yang, Yuyang Jia, Minwen Zheng

**Affiliations:** ^1^Department of Radiology, Xijing Hospital, Fourth Military Medical University, Xi'an, China; ^2^Siemens Healthineers Ltd., Shanghai, China; ^3^Department of Cardiology, Xijing Hospital, Fourth Military Medical University, Xi'an, China

**Keywords:** pericoronary adipose tissue, pericoronary adipose tissue CT attenuation, radiomics analysis, ischemic coronary artery stenosis, fractional flow reserve

## Abstract

**Objective:**

To investigate the influence of different segmentations on the diagnostic performance of pericoronary adipose tissue (PCAT) CT attenuation and radiomics features for the prediction of ischemic coronary artery stenosis.

**Methods:**

From June 2016 to December 2018, 108 patients with 135 vessels were retrospectively analyzed in the present study. Vessel-based PCAT was segmented along the 40 mm-long proximal segments of three major epicardial coronary arteries, while lesion-based PCAT was defined around coronary lesions. CT attenuation and radiomics features derived from two segmentations were calculated and extracted. The diagnostic performance of PCAT CT attenuation or radiomics models in predicting ischemic coronary stenosis were also compared between vessel-based and lesion-based segmentations.

**Results:**

The mean PCAT CT attenuation was −75.7 ± 9.1 HU and −76.1 ± 8.1 HU (*p* = 0.395) for lesion-based and vessel-based segmentations, respectively. A strong correlation was found between vessel-based and lesion-based PCAT CT attenuation for all cohort and subgroup analyses (all *p* < 0.01). A good agreement for all cohort and subgroup analyses was also detected between two segmentations. The diagnostic performance was comparable between vessel-based and lesion based PCAT CT attenuation in predicting ischemic stenosis. The radiomics features of PCAT based on vessel or lesion segmentation can both adequately identify the ischemic stenosis. However, no significant difference was detected between the two segmentations.

**Conclusions:**

The quantitative evaluation of PCAT can be reliably measured both from vessel-based and lesion-based segmentation. Furthermore, the radiomics analysis of PCAT may potentially help predict hemodynamically significant coronary artery stenosis.

## Introduction

Vascular inflammation is a driver of coronary atherosclerotic plaque formation and also a typical feature of atherosclerotic plaque rupture (1). Patients with coronary artery disease (CAD) have histological evidence of local inflammation both within culprit lesions and throughout the entire coronary vascular bed (2). Coronary computed tomography angiography (CCTA) is a widely used non-invasive modality for the diagnosis of CAD. Recent research demonstrated that signals released from the inflamed coronary artery diffuse to the perivascular adipose tissue, inhibiting local adipogenesis. Such an inflammatory response changes the composition of perivascular adipose tissue around inflamed arteries, shifting its attenuation on CCTA from the lipid [more negative Hounsfield unit (HU) values (e.g., closer to −190 HU)] to the aqueous phase [less negative HU values (e.g., closer to −30 HU)]. The changes in pericoronary adipose tissue (PCAT) attenuation can be non-invasively measured using routine CCTA, and enable early detection of vascular inflammation in coronary arteries. The relationship between PCAT and coronary atherosclerosis has been studied by several authors, including its link with plaque composition, vulnerability, and hemodynamic significance (3–6). Moreover, higher PCAT CT attenuation was revealed to be associated with an increased risk of cardiac mortality and poor prognosis (7). The importance of PCAT radiomics features in cardiac risk prediction was also revealed by Oikonomou et al. (8). However, the area of PCAT measurement in different studies was inconsistent. Among previous studies, the majority investigated PCAT in the proximal 40-mm segment of all three major epicardial coronary vessels [right coronary artery (RCA), left anterior descending artery (LAD), and left circumflex artery (LCX)] (5, 7, 9–11) or one of the coronary arteries (RCA or LAD) (2–4, 8, 12–16). Some studies suggested that a lesion-specific assessment of PCAT might provide greater insight into atherosclerotic biology than the proximal segments of the major arteries alone (6, 17). To resolve this inconsistency, this study aimed to clarify whether a proximal 40 mm assessment or a lesion-specific assessment was more appropriate in the evaluation of PCAT. Our secondary objective was to compare the diagnostic performance of PCAT CT attenuation and radiomics features between two segmentations for the prediction of hemodynamically significant coronary artery stenosis.

## Methods

### Study Patients

This retrospective study complied with the Helsinki Declaration (2000). From June 2016 to December 2018, 191 consecutive patients with suspect CAD who underwent CCTA, invasive coronary angiography (ICA), and fractional flow reserve (FFR) examination were retrospectively screened from our institution's database. All patients gave written informed consent, and the study protocol was approved by the institutional review board of Xijing Hospital affiliated with the Fourth Military Medical University (KY20194007). Exclusion criteria were previous revascularization (*n* =47), normal angiograms in CCTA (*n* = 1), the interval between CCTA and FFR measurement > 1 month (*n* = 28) and poor CCTA image quality (*n* =7). Additionally, vessels with total occlusion (*n* = 8) and normal angiogram (*n* = 5) were further excluded (Supplementary Figure S1). Finally, 135 lesions in 108 patients (mean age, 59 years ± 10; range, 30 ~ 77 years, 78 males and 20 females) were analyzed. Details of the patients' characteristics were shown in Table 1.

**Table 1:** Patients characteristics.

**Characteristics**	**Overall (***n*** = 108)**
Age, mean (SD), years	59 ± 10
Male, *n* (%)	78 (72.2%)
Body mass index, mean (SD), kg/m^2^	24.8 ± 2.9
Risk factors, *n* (%)	
Diabetes mellitus	11 (10.2%)
Hypertension	54 (50.0%)
Hyperlipidemia	38 (35.2%)
Current smoker	45 (41.6%)
CACS, Agatston	188.8 (54.1–398.12)
Interval between ICA and CCTA, mean (SD), days	9 ± 6

*CACS, coronary artery calcium score; CCTA, coronary computed tomography angiography; ICA, invasive coronary angiography; SD, Standard deviation*.

### Image Acquisition and Analysis

All CCTA scans were performed on a second generation 128-slice dual source CT (Somatom Definition Flash, Siemens Healthineers, Forchheim, Germany) with retrospectively electrocardiogram-triggered spiral acquisition. The detailed scanning parameters are specified in Supplementary Appendix. Plaque characterization was performed using dedicated plaque analysis software (Coronary Plaque Analysis, version 5.0.0, Siemens Healthineers, Germany). Coronary artery calcium score was calculated on gated non-contrast CT using the Agatston method.

### ICA and FFR Measurement

Selective ICA was performed by standard catheterization in accordance with the American College of Cardiology recommendations for coronary angiography (18). FFR was measured using a 0.014-inch pressure sensor tipped guidewire (Pressure Wire, St. Jude Medical Systems, St. Paul, Minnesota) as previously described (19). Hyperemia was induced with intravenous continuous infusion (160 μg/kg/min) of adenosine (20). Intracoronary nitroglycerin was administered immediately before measurement of FFR. FFR ≤ 0.80 was indicative of hemodynamically significant stenosis.

### PACT Analysis

**Figure 1:**
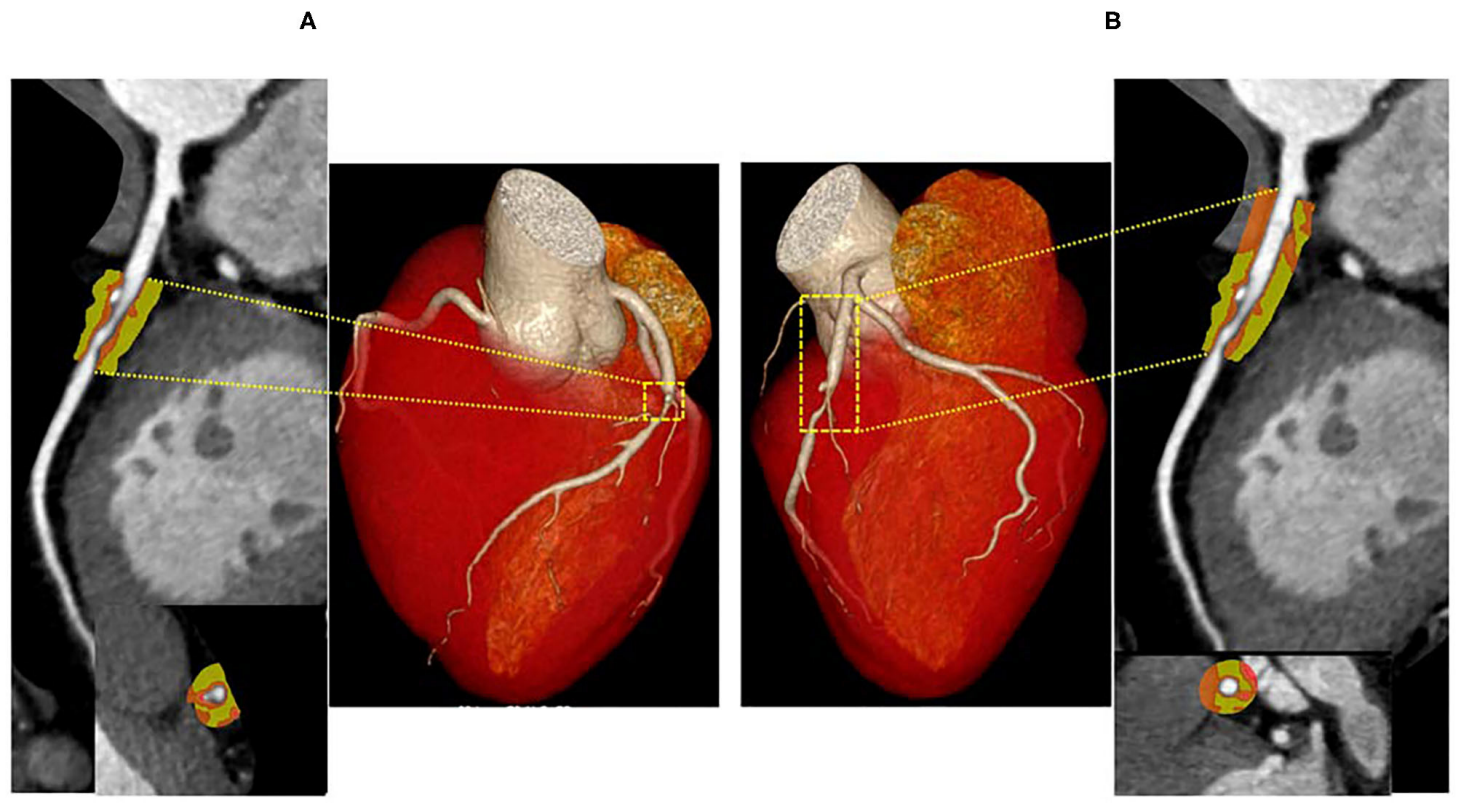
Representative CCTA image of PCAT around LAD. A hemodynamically significant stenosis (FFR = 0.73) in the middle segment of LAD. PCAT CT attenuation was defined as the mean CT attenuated value within a radial distance equal to the diameter of the vessel. The length of the lesion-based PCAT was defined from the proximal to the distal shoulder of the lesion **(A)**, the vessel-based PCAT was focused on the proximal 40-mm segments of all three major epicardial coronary vessels (10 to 50 mm from right coronary artery ostium) **(B)**. LAD, left anterior descending artery; PCAT, pericoronary adipose tissue.

To measure PCAT CT attenuation, 3-dimensional layers within radial distance from the outer coronary wall equal in thickness to the average diameter of the vessels were constructed automatically from the CCTA (Figure 1) using semi-automated software (Perivascular Analysis function, Coronary Plaque Analysis, version 5.0.0, Siemens Healthineers, Germany). Within the predefined volume of interest, voxels with tissue attenuation ranging from −190 HU to −30 HU were defined as PCAT (7). Two cardiovascular radiologists (with 8- and 10-years of experience in cardiac imaging) who were blinded to ICA and FFR results, independently performed the PCAT segmentation. On a per vessel level, PCAT segmentation was performed around the 40 mm-long proximal segments of LAD, LCX, and RCA. To avoid the effects of the aortic wall, we excluded the most proximal 10 mm of RCA and analyzed the proximal 10–50 mm of the vessel (7). In LAD and LCX, we did not analyze the left main coronary artery because of its variable length. Given that previous studies (6, 17) have shown PCAT surrounding lesions to be a potential sensor of ischemic stenosis, lesion-based PCAT segmentation was also performed around coronary lesions on a per lesion level. The lesion causing the highest-grade stenosis on each coronary vessel was chosen for PCAT analysis, and the length of the lesion-based PCAT was defined from the proximal to the distal shoulder of the lesion. PCAT CT attenuation was defined as the mean CT attenuation in the adipose tissue.

### Radiomics Analysis

Lesion- and vessel-based PCAT segmentations were loaded into a stand-alone software prototype (Radiomics, version 1.2.2, Siemens Healthineers, Germany), which extracted radiomics features via PyRadiomics library. A total of 1,691 features were calculated for each segmentation, including 17 shape, 18 first-order and 75 texture features in the original images. High-dimensional radiomics features were calculated through pre-processing, which multiplied first-order and texture features through Laplacian of Gaussian (LoG) filtering, wavelet filtering, and non-linear intensity transforms (Supplementary Table S1).

### Radiomics Models Construction

Eligible lesions were randomly split into a training (60%) and a testing set (40%), in order to identify optimal radiomics features extracted from different PCAT segmentations and to further validate their association with hemodynamic status of coronary artery stenosis. Univariate logistic regression was conducted in the training dataset to initially screen out radiomics features irrelevant to hemodynamic outcome by creating Manhattan plots. Based on a pre-defined statistical significance of *p* < 0.05, features above the threshold of 1.301 (–log10 based) remained for more rigorous selection. The least absolute shrinkage and selection operator (LASSO) with logistic regression was used to separately select the ideal combination of features from lesion-based and vessel-based PCAT. Finally, 10-fold cross validation was applied to determine the optimal lambda value, which was fed into the prediction model in order to discern dichotomized hemodynamic significance.

### Statistical Analysis

Continuous variables are presented as mean ± standard deviation and categorical variables as frequencies (percentages). The interobserver agreement for PCAT CT attenuation was determined with the intraclass correlation coefficient (ICC). Pearson correlation coefficient was calculated for assessment of the relationship of vessel-based and lesion-based PCAT CT attenuation in the entire cohort as well as sub-group analysis. Bland-Altman plots were created comparing vessel-based and lesion-based PACT CT attenuation. Confidence limits were created assuming a parametric distribution, and 1-sample *t*-tests were run to detect any significant fixed bias. Diagnostic performance of PCAT radiomics features was assessed with receiver operating characteristic (ROC) curve analysis, and the DeLong test was used to compare the area under the curves (AUCs). Statistical analysis was performed using MedCalc version 11.4.2 (MedCalc Software, Mariakerke, Belgium) and R, version 3.6.3 (R Foundation for Statistical Computing, Vienna, Austria). A 2-tailed value of *p* < 0.05 was considered statistically significant.

## Results

### Lesion Characteristics

Lesions were most often present in LAD (71.8%), followed by RCA (15.6%) and LCX (12.6%). Among the 135 lesions, 76 (56.3%) lesions were located in the proximal of coronary artery, 33 (24.4%) lesions in the middle segment and 26 (19.3%) lesions in the distal segment. Moreover, 103 (76.3%) lesions with stenosis ≥50% were detected, and 63 (46.7%) lesions were considered ischemic stenosis according to FFR (FFR ≤ 0.80) (Table 2). The mean lesion length was 26.2 ± 12.3 mm.

**Table 2:** Lesion characteristics in coronary computed tomography angiography.

**Variables**	**Overall (***n*** = 135)**	**FFR > 0.80 (***n*** = 72)**	**FFR ≤0.80 (***n*** = 63)**	***p***
DS (%), mean (SD)	61% ± 19%	59% ± 21%	64% ± 17%	0.084
Lesion localization, *n* (%)				0.000
LAD	97 (71.8%)	39 (28.9%)	58 (43.0%)	
LCX	17 (12.6%)	15 (11.1%)	2 (1.5%)	
RCA	21 (15.6%)	18 (13.3%)	3 (2.2%)	
Lesion territory				0.046
Proximal	76 (56.3%)	47 (34.8%)	29 (21.5%)	
Middle	33 (24.4%)	12 (8.9%)	21 (15.6%)	
Distal	26 (19.6%)	13 (9.6%)	13 (9.6%)	
Lesion length, mean (SD), mm	26.2 ± 12.3	23.2 ± 11.5	29.6 ± 12.4	0.002
MLA, mean (SD), mm^2^	3.5 ± 2.4	4.0 ± 2.7	2.9 ± 1.7	0.007
Total plaque volume (mm^3^)	223.4 ± 191.1	214.7 ± 182.8	233.3 ± 201.0	0.452
Calcified plaque volume (mm^3^)	76.8 ± 111.84	70.4 ± 109.1	84.0 ± 115.3	0.519
Fibrotic plaque volume (mm^3^)	139.4 ± 94.41	136.7 ± 91.4	142.5 ± 98.3	0.685
Lipid plaque volume (mm^3^)	7.2 ± 12.4	7.7 ± 13.3	6.8 ± 11.4	0.654
PCAT CT attenuation, mean (SD), HU				
Lesion-based segmentation	−75.7 ± 9.1	−75.1 ± 9.9	−76.3 ± 8.3	0.463
Vessel-based segmentation	−76.1 ± 8.1	−76.1 ± 7.9	−75.5 ± 8.5	0.453

*DS, diameter stenosis; FFR, fractional flow reserve; LAD, left coronary artery; LCX, left circumflex; MLA, minimal lumen area; PCAT, pericoronary adipose tissue; RCA, right coronary artery; SD, Standard deviation*.

### PCAT CT Attenuation

**Figure 2:**
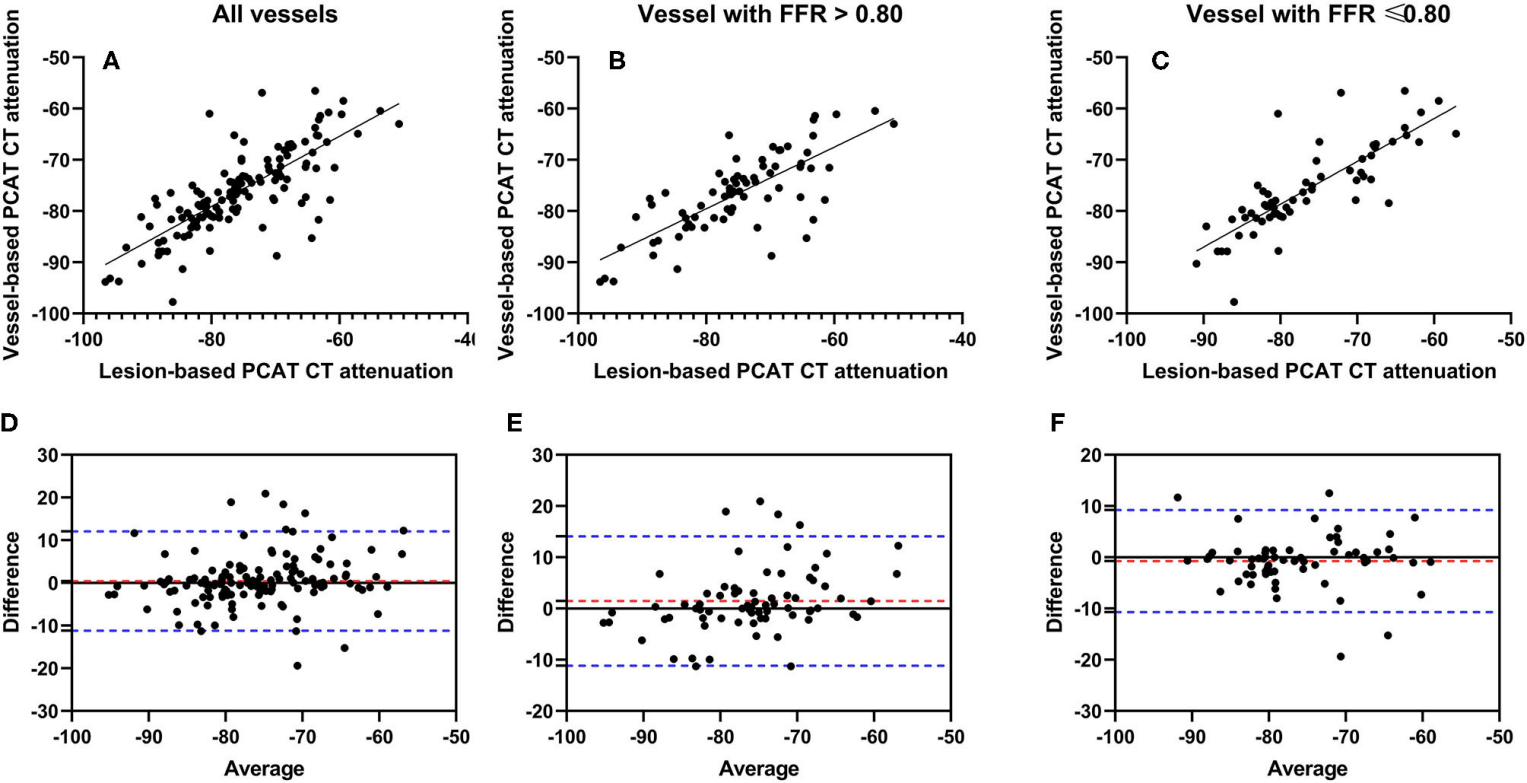
Correlation and Bland-Altman plots of vessel-based and lesion-based PCAT CT attenuation as clarified by invasive FFR. Pearson correlation analyses show the correlation vessel-based and lesion-based PCAT CT attenuation in all vessels **(A)**, vessels with FFR > 0.80 **(B)** and vessels with FFR ≤ 0.80 **(C)**, respectively. Bland-Altman plots show PCAT CT attenuation between vessel-based and lesion-based in all vessels **(D)**, vessels with FFR >0.80 **(E)** and vessels with FFR ≤ 0.80 **(F)**. The red dotted lines represent the mean difference (bias), and the blue dotted lines represent the 95% limits of agreement. FFR, fractional flow reserve; PCAT, pericoronary adipose tissue.

PCAT CT attenuation on lesion-based and vessel-based segmentation had excellent interobserver reproducibility with ICC of 0.921 and 0.984 in all vessels, 0.918 and 0.982 in vessels with FFR >0.80, and 0.932 and 0.991 in vessels with FFR ≤ 0.80. The mean PCAT CT attenuation was −75.7 ± 9.1 HU for lesion-based measurement, and −76.1 ± 8.1 HU for vessel-based measurement (*p* = 0.395) (Table 2). A strong significant correlation was observed between vessel-based and lesion-based PCAT CT attenuation (*r* = 0.7686, *p* < 0.0001) in the entire cohort (Figure 2A), as well as sub-group analysis [*r* = 0.7540, *p* < 0.0001 for vessels with FFR > 0.80 (Figure 2B) and *r* = 0.8166, *p* < 0.0001 for vessels with FFR ≤ 0.80 (Figure 2C)]. A Bland-Altman analysis comparing lesion-based and vessel-based PCAT CT attenuation showed the following mean differences: 0.4356 (95% limits of agreement: −11.18 to 12.05) in all vessels (Figure 2D), 1.467 (−11.16 to 14.09) for vessels with FFR >0.80 (Figure 2E) and −0.743 (−10.70 to 9.212) for vessels with FFR ≤ 0.80 (Figure 2F), respectively.

**Figure 3:**
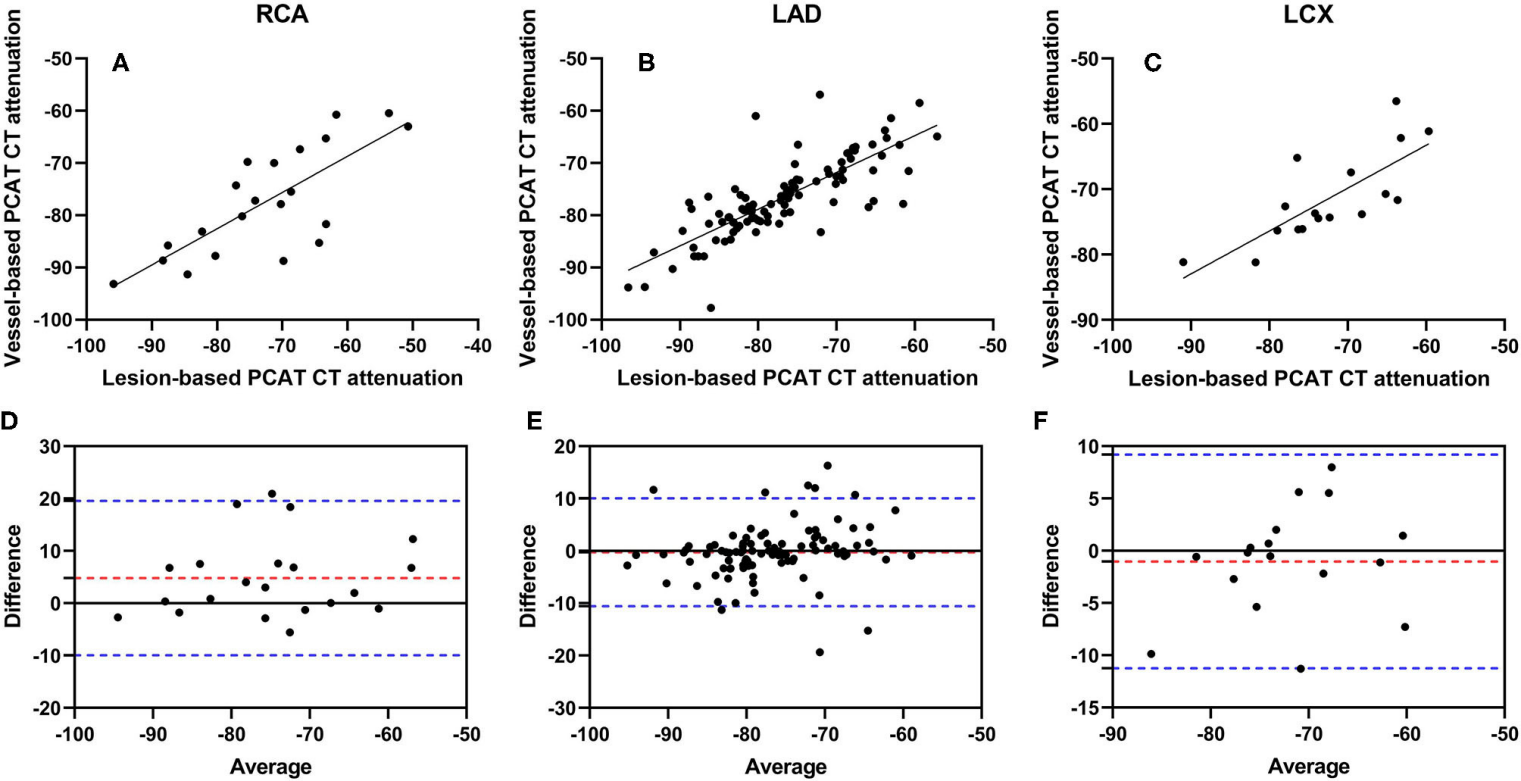
Correlation and Bland-Altman plots of vessel-based and lesion-based PCAT CT attenuation in different coronary arteries. Pearson correlation analyses show the correlation vessel-based and lesion-based PCAT CT attenuation in RCA **(A)**, LAD **(B)** and LCX **(C)**, respectively. Bland-Altman plots show PCAT CT attenuation between vessel-based and lesion-based in RCA **(D)**, LAD **(E)** and LCX **(F)**. The red dotted lines represent the mean difference (bias), and the blue dotted lines represent the 95% limits of agreement. LAD, left anterior descending artery; LCX, left circumflex artery; PCAT, pericoronary adipose tissue; RCA, right coronary artery.

Pearson correlation coefficients of vessel-based and lesion-based PCAT CT attenuation were as follows: RCA: *r* = 0.7649 (*p* = 0.0001) (Figure 3A), LAD: *r* = 0.7921 (*p* < 0.0001) (Figure 3B), and LCX: *r* = 0.7656 (*p* = 0.0003) (Figure 3C). The difference between vessel-based and lesion-based PCAT CT attenuation in RCA was 4.821 (95% limits of agreement: −9.928 to 19.57) (Figure 3D), −0.2577 (−10.56 to 10.05) in LAD (Figure 3E), and −1.026 (−11.25 to 9.195) in LCX (Figure 3F). Furthermore, a good correlation and agreement of vessel-based and lesion-based PCAT CT attenuation was found regardless of lesion locations (Supplementary Figure S2).

**Figure 4:**
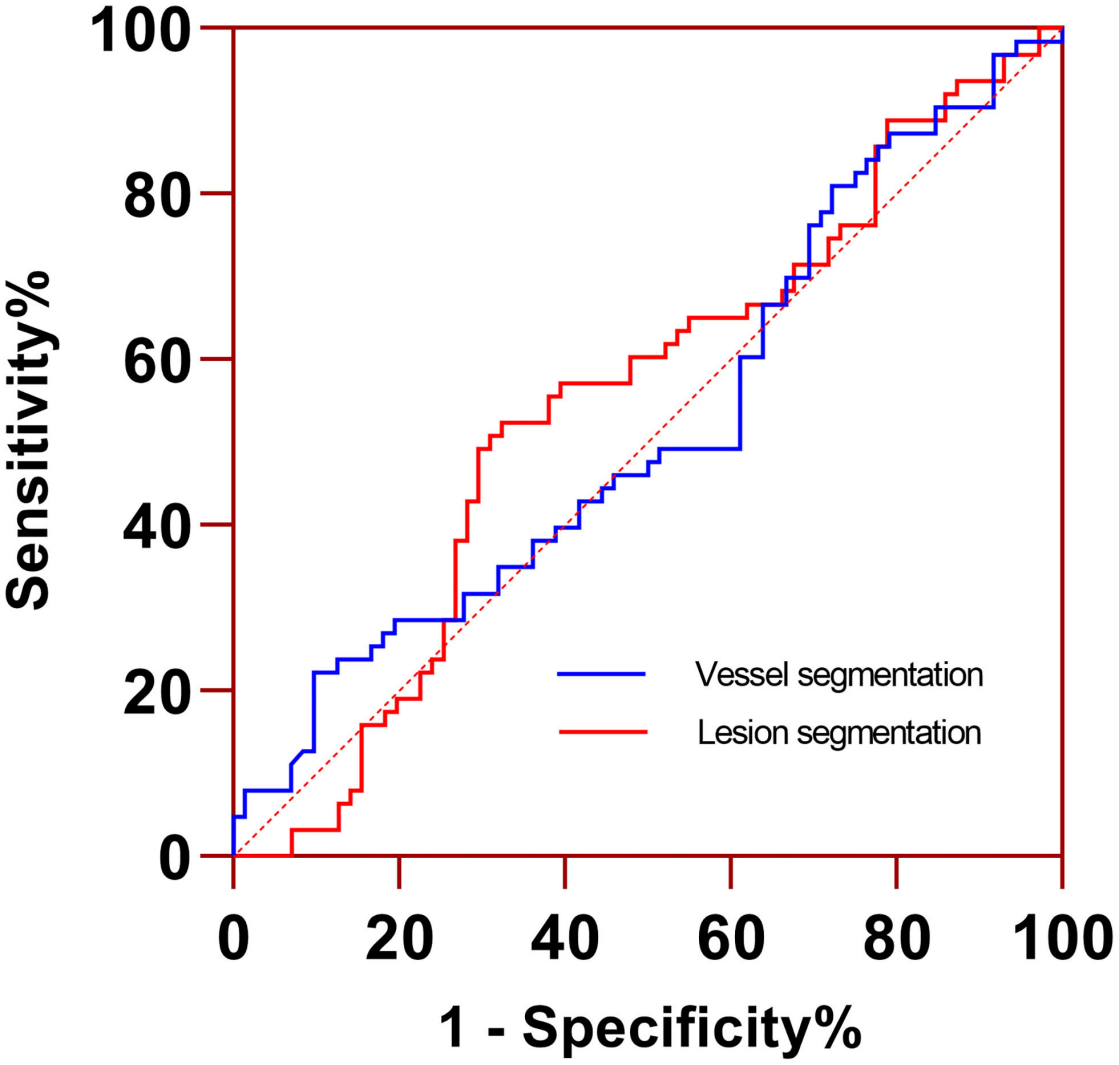
ROC curve analysis of PCAT CT attenuation measured from lesion- and vessel-based segmentations for identifying functionally significant coronary stenosis. Two segmentations had similar AUC (0.547 vs. 0.524, *p* = 0.814). AUC, area under the curve; ROC, receiver operating characteristic curve.

There was no significant difference in PCAT CT attenuation between vessels with FFR >0.80 and FFR ≤ 0.80 regardless of the segmentation method (vessel-based *p* = 0.453; lesion-based *p* = 0.463). According to ROC curve analysis, the AUC of PCAT CT attenuation in vessel-based segmentation was 0.524 (95% CI: 0.436–0.611), which had similar diagnostic performance compared to lesion-based PCAT CT attenuation (0.547, 95% CI: 0.459–0.633, *p* = 0.814) (Figure 4).

### Radiomics Features of PCAT

**Figure 5:**
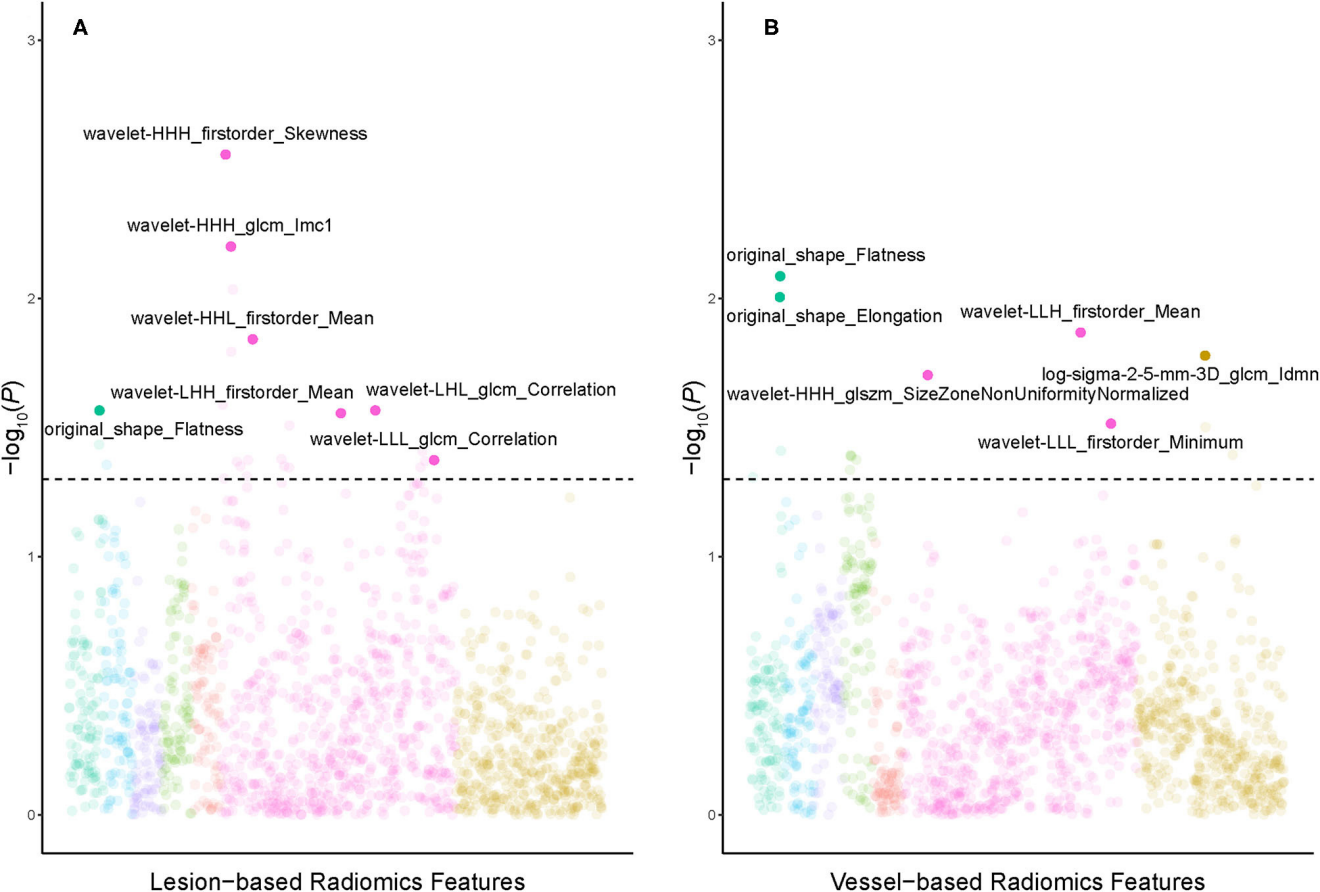
Manhattan plots demonstrated the strength of association [–log10 (*P*-value)] of univariate logistic regression] comparing PCAT radiomics features derived from lesion-based **(A)** and vessel-based **(B)** PCAT segmentation against the hemodynamic significance of coronary stenosis. Solid points above the horizontal cutoff line denote features qualified for univariate logistic regression and LASSO-logistic regression, and their corresponding feature names are displayed. LASSO, least absolute shrinkage and selection operator; PCAT, pericoronary adipose tissue.

The dataset was randomly split into a training set (*n* = 82) and a testing set (*n* = 53). There was no difference in stenosis diameter, lesion distribution, and PCAT CT attenuation between the training and the testing set (all *p* > 0.05) (Table 3). Univariable logistic regression selected 24 and 15 radiomics features from lesion-based and vessel-based PCAT segmentations, respectively. Ten-fold cross validation was performed to further refine the remaining features. Seven features from lesion-based PCAT segmentation (Figure 5A), and six from vessel-based PACT segmentation (Figure 5B) were ultimately used to construct the radiomics models using LASSO.

**Table 3:** Lesion characteristics in the training and testing cohorts.

**Variables**	**Overall (***n*** = 135)**	**Training set (***n*** = 82)**	**Testing set (***n*** = 53)**	***p***
DS (%), mean (SD)	61.8% ± 18.6%	63.3% ± 18.5%	59.4% ± 18.5%	0.239
Lesion localization, *n* (%)				0.395
LAD	97 (71.9%)	62 (45.9%)	35 (25.9%)	
LCX	17 (12.6%)	8 (5.9%)	9 (6.6%)	
RCA	21 (15.6%)	12 (8.9%)	9 (6.7%)	
Lesion territory				0.856
Proximal	76 (56.3%)	45 (33.3%)	31 (23.0%)	
Middle	33 (24.4%)	20 (14.8%)	13 (9.6%)	
Distal	26 (19.3%)	17 (12.6%)	9 (6.7%)	
Lesion length, mean (SD), mm	26.1 ± 12.3	37.6 ± 12.6	24.0 ± 11.7	0.102
MLA, mean (SD), mm^2^	3.5 ± 2.4	3.5 ± 2.4	3.5 ± 2.3	0.913
Total plaque volume (mm^3^)	223.4 ± 191.1	238.5 ± 194.8	199.7 ± 184.4	0.254
Calcified plaque volume (mm^3^)	76.8 ± 111.8	82.1 ± 107.0	68.5 ± 119.7	0.496
Fibrotic plaque volume (mm^3^)	139.4 ± 94.4	148.9 ± 99.8	124.4 ± 84.0	0.144
Lipid plaque volume (mm^3^)	7.2 ± 12.4	7.5 ± 13.5	6.8 ± 10.5	0.759
PCAT CT attenuation, mean (SD), HU				
Lesion-based segmentation	−75.7 ± 9.1	−76.1 ± 9.5	−75.0 ± 8.6	0.518
Vessel-based segmentation	−76.1 ± 8.1	−76.2 ± 8.6	−76.0 ± 7.5	0.881
FFR	0.809 ± 0.007	0.808 ± 0.079	0.811 ± 0.086	0.969

*DS, diameter stenosis; FFR, fractional flow reserve; HRP, high risk plaque; LAD, left coronary artery; LCX, left circumflex; MLA, minimal lumen area; PCAT, pericoronary adipose tissue; RCA, right coronary artery; SD, Standard deviation*.

**Figure 6:**
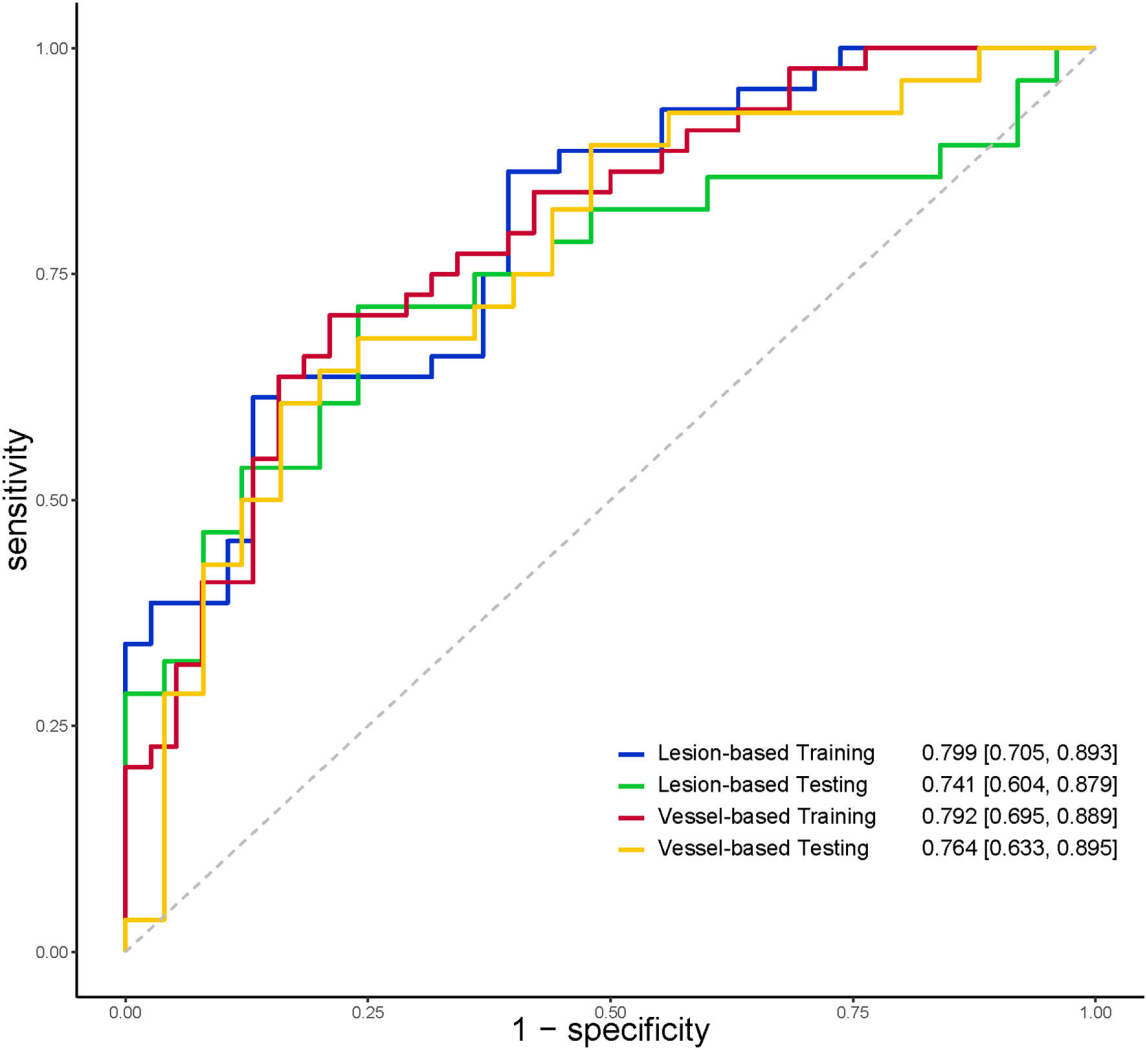
ROC curves indicated comparable diagnostic performance between vessel-based and lesion-based PCAT radiomics models for identifying functionally significant coronary stenosis. PCAT, pericoronary adipose tissue; ROC, receiver operating characteristic.

The discriminatory power of radiomics models was shown in Table 4. The AUC, specificity, and sensitivity of the radiomics model of lesion-based PCAT segmentation in the training set were 0.799, 86.8%, and 61.4%, 0.741, 80.0%, and 53.6% in the testing set, respectively (Figure 6). The vessel-based radiomics model produced comparable performance: 0.792, 78.9, and 70.5% in the training set, and 0.763, 64.0, and 67.9% in the testing set. No significant difference was detected between vessel-based and lesion-based PCAT segmentations for the prediction of ischemic coronary artery stenosis in both the training set (AUC: 0.792 vs. 0.799, *p* = 0.900) and the testing set (AUC: 0.764 vs. 0.741, *p* = 0.810).

**Table 4:** Discriminatory power of radiomics models.

**Different segmentations**	**AUC (95% CI)**	**Sensitivity**	**Specificity**	**Accuracy**
Training set (*n* = 82)				
Vessel-based segmentation	0.792 (0.695–0.889)	0.705	0.789	0.744
Lesion-based segmentation	0.799 (0.705–0.893)	0.614	0.868	0.732
*p*	0.900			
Testing set (*n* = 53)				
Vessel-based segmentation	0.764 (0.633–0.895)	0.679	0.640	0.660
Lesion-based segmentation	0.741 (0.604–0.879)	0.536	0.800	0.660
*p*	0.810			

## Discussion

The current study compared PCAT CT attenuation and radiomics features derived from different PCAT segmentations. Our study's main finding is that vessel-based segmentation shows good agreement with lesion-based segmentation, along with a small bias for PCAT CT attenuation. In addition, vessel-based and lesion-based PCAT radiomics models show comparable diagnostic performance in predicting ischemic stenosis.

Most studies (3–5, 7, 10, 13, 21) have validated that higher vessel-based PCAT CT attenuation was associated with an increased risk of cardiac mortality, poor prognosis, higher plaque burden and impaired myocardial perfusion. However, the association between vessel-based PCAT CT attenuation and the hemodynamic stenosis was not reported. A recent published study (6) demonstrated that the increase of lesion-based PCAT CT attenuation was associated with the hemodynamic significant of stenosis. Our study evaluated both lesion-based and vessel-based PCAT CT attenuation, as well as their respective diagnostic performance for the hemodynamic stenosis. The present results showed PCAT CT attenuation derived from vessel-based and lesion-based segmentations had a good agreement for all vessels analysis as well as sub-group analysis. However, the good correlation and agreement of PCAT CT attenuation between lesion-based and vessel-based segmentation in our study may be attributed to the location and length of the lesion. In this study, 80.7% (109/135) of lesions were located in the proximal to middle segments of coronary artery, with the mean lesion length of 26.2 ± 12.3 mm. In view of good correlation between PCAT CT attenuation measurements from vessel segmentation and lesion segmentation, therefore, quantitative evaluation of PCAT can be reliable from both segmentations. As with real-world studies, the PACT segmentation can be at the discretion of the radiologists.

However, it was unexpected that PCAT CT attenuation was not significantly different between hemodynamically significant stenoses and non-significant ones, regardless of the PCAT segmentation method. This finding is inconsistent with two previous studies (6, 16). The mean PCAT CT attenuation in coronary arteries with FFR >0.80 was similar to the above-mentioned studies (6, 16). The discrepancy in PCAT CT attenuation between this study and the previous studies was predominantly in coronary arteries with FFR ≤ 0.80. The probable explanation may be attributed to the different diameter stenosis. Similar to previous studies (5, 16), patients with high PCAT CT attenuation usually had high-grade stenosis according to previous studies. In our clinical practice, most patients referred for FFR often had intermediate stenosis (30–70%). The mean stenosis diameter (64.0 ± 17.3%) in coronary arteries with FFR ≤ 0.80 was obviously less severe in our study than in the Yu et al. study (76.32 ± 8.52%) (6); thus, leading to a low mean PCAT CT attenuation. Statistical differences in degree of stenosis between coronary arteries with FFR >0.80 and those with FFR ≤ 0.80 did differ between Yu et al. (6) and our study, however, the diagnostic performance of PCAT CT attenuation in predicting ischemic stenosis in Yu et al. was slightly superior than in our study (AUC: 0.630 vs. 0.547 and 0.524). In the Hoshino et al. study, several patients with severe stenosis (FFR < 0.5) with elevated PCAT CT attenuation were enrolled, thus leading to a higher average PCAT CT attenuation. However, patients enrolled in the present study were hemodynamically less severe, which is reflected in the minimal FFR value of 0.59. Thus, the mean PCAT CT attenuation in coronary arteries with FFR ≤ 0.80 was lower, and not significantly different from those with FFR >0.80.

PCAT analysis was also performed around either all three major epicardial coronary vessels (5, 7, 9–11) or one of the coronary arteries (RCA or LAD) (2–4, 8, 12–16). The CRISP CT showed the statistical collinearity between PCAT CT attenuation measurements around RCA and LAD. Our study extended earlier findings and indicated that the high correlation between vessel-based and lesion-based PCAT CT attenuation was also detected in all three major epicardial coronary vessels.

Radiomics analysis suggested that no significant differences were found between lesion-based and vessel-based radiomics models with comparable AUCs (0.799 vs. 0.792 in training set, and 0.741 vs. 0.764 in testing set). Radiomics features were mainly limited to intensity and texture, although the detailed features were not identical in the two segmentation methods. Among the significant radiomics features, texture homogeneity and wavelet-transformed intensity distribution within PCAT were particularly important in predicting the hemodynamic significance of coronary stenosis, regardless of different segmentation methods. A possible reason is that coronary stenoses are usually followed by an inflammatory response, which stimulates neovascularization and fibrosis while breaking down PCAT (8). As a result of lipolysis, local inflammation leads to sporadic transformation of adipose tissue to an aqueous component, hence increasing CT attenuation and tissue heterogeneity. Therefore, intensity and texture radiomics features could be indications of such processes, which suggest the potential development of coronary stenoses.

Geometric characteristics such as flatness and elongation were also highlighted in our study, although PCAT was segmented with strictly defined rules. These metrics are defined as the square root of the ratio between the lesser and the largest principal components in a three-dimensional volume. In the vessel-based segmentations with constant length, elongation and flatness depend mainly on the stiffness of vessels. Vessels with flow-limiting (FFR ≤ 0.80) lesions were associated with impaired vasodilator capacity, and were likely stiffer than vessels with non-flow-limiting lesions (6). The visually indiscernible slight change of geometric features of PCAT adjacent to vessel wall could be captured by radiomics. Therefore, the two geometric radiomics features of elongation and flatness may be associated with FFR.

## Limitations

This study had some limitations. Firstly, this was a single-center, retrospective study with a relatively small sample size. An inclusion bias existed because the study population did not include patients with minimal or severe stenosis. Furthermore, due to its retrospective nature, the impact of the related clinical factors on PCAT CT attenuation, such as serum highly sensitive C-reactive protein level, left ventricular ejection fractions, left ventricular hypertrophy, etc., could not be investigated in our study. Secondly, all patients underwent CCTA using the same CT scanner and protocol. Hence, the generalizability of our findings to other populations may be limited, as image acquisition and reconstruction settings can affect the reproducibility of PCAT CT attenuation and radiomics features. Thirdly, the software for PCAT quantification is limited to a single vendor and is not yet commercially available. Finally, we did not exclude the patients treated by statins in this study, and potentially introduced unnecessary bias to our results as statins may influence PCAT CT attenuation and PCAT radiomics.

## Conclusions

This study demonstrated that quantitative evaluation of PCAT can be reliably measured both from lesion-based and vessel-based segmentation. We also found that PCAT radiomics features may potentially help predict hemodynamically significant coronary stenoses.

## Data Availability Statement

The original contributions presented in the study are included in the article/Supplementary Material, further inquiries can be directed to the corresponding author.

## Ethics Statement

The studies involving human participants were reviewed and approved by Xijing Hospital of Fourth Military Medical University. The patients/participants provided their written informed consent to participate in this study.

## Author Contributions

MZ: guarantor of integrity of the entire study. DW and MZ: study concepts and design. RA: literature research. WY and RA: clinical studies. SL and YJ: experimental studies and data analysis. DW and SL: statistical analysis. DW and RA: manuscript preparation. RA and MZ: manuscript editing. All authors contributed to the article and approved the submitted version.

## Funding

This study has received funding from the National Natural Science Foundation of China (Grant No. 82071917 to MZ), the Key Research and Development Plan of Shaanxi Province (Grant No. 2020ZDLSF01-01 to MZ), the National Science Foundation of Shaanxi Province (Grant No. 2020JQ-461 to DW), and the Discipline Promotion Projects of Xijing Hospital (Grant No. XJZT19Z13 to MZ).

## Conflict of Interest

SL was employed by the company Siemens Healthineers Ltd. The remaining authors declare that the research was conducted in the absence of any commercial or financial relationships that could be construed as a potential conflict of interest.

## Publisher's Note

All claims expressed in this article are solely those of the authors and do not necessarily represent those of their affiliated organizations, or those of the publisher, the editors and the reviewers. Any product that may be evaluated in this article, or claim that may be made by its manufacturer, is not guaranteed or endorsed by the publisher.
