# Supplementary material for: Influence of Different Segmentations on the Diagnostic Performance of Pericoronary Adipose Tissue
Source: Front Cardiovasc Med. 2022 Mar 3;9:773524. doi: 10.3389/fcvm.2022.773524 (PMC8929663; doi:10.3389/fcvm.2022.773524)

**Supplemental Material**

**Influence of different segmentations on the diagnostic performance of pericoronary adipose tissue**

Didi Wen^1^, Rui An^1^, Shushen Lin^2^, Wangwei Yang^3^, Yuyang Jia^1^, Minwen Zheng^1^

^1^Department of Radiology, Xijing Hospital, Fourth Military Medical University, 127# Changle West Road, Xi’an 710032, China

^2^Siemens Healthineers Ltd., 399 West Haiyang Road, Shanghai 200124, China

^3^Department of Cardiology, Xijing Hospital, Fourth Military Medical University, 127# West Changle Road, Xi’an 710032, China

**Supplemental methods**

**Image Acquisition**

Retrospectively ECG-triggered spiral acquisition of CCTA scanning was performed on a second generation 128-slice dual source CT (Somatom Deﬁnition Flash, Siemens Healthineers, Forchheim, Germany). Oral β-blocker 50 mg (Metoprolol; Betaloc, AstraZeneca, Cambridge, England) was administered to patients with a heart rate ≥ 70 beats/min. Each patient received a sublingual dose of isosorbide dinitrate 2.5 mg (Isoket; Schwarz Pharma, Monheim, Germany) 2 minutes before image acquisition.

The CCTA scanning parameters were as follows: a pitch of 0.2-0.5 adapted to the heart rate, slice collimation 2 × 128 × 0.6 mm by means of z-flying focal spot, tube voltage 100 kV, reference tube current 300 mAs with automatic tube current modulation, gantry rotation time 0.28 s and the image acquisition range was from 2 cm below the bifurcation of trachea to the diaphragm.

An intravenous bolus of 1 ml/kg Iopromide 370 (370 mg I/ml, Ultravist 370, Bayer Schering Pharma, Berlin, Germany) was injected at a flow rate of 5 ml/s followed by 40 ml saline solution. Contrast agent application was controlled by bolus tracking. The region of interest (ROI) was placed into the aortic root, and image acquisition started 5 s after the signal attenuation reached the predefined threshold of 100 Hounsfield units (HU).

Data were transferred to an offline workstation (Syngo.Via; Siemens Healthineers) for further analysis. Axial, cross-sectional, curved planar reformation, multiplanar reformation, and three-dimensional maximum intensity projection images were generated.

**Supplemental Tables**

**Table S1** Radiomics Features Composition

|  | **Original** | **LoG filtering** | **Wavelet filtering** | **Non-linear intensity transforms** | **Total** |
| --- | --- | --- | --- | --- | --- |
|  |  | 0.5, 1.5, 2.5, 3.5, 4.5 mm | LLL, LLH, LHL, LHH, HLL, HLH, HHL, HHH | square, square root, logarithm, exponential |  |
| **Shape** | 17 |  |  |  | 17 |
| **First-order** | 18 | 18 × 5 | 18 × 8 | 18 × 4 | 324 |
| **Texture** | 75 | 75 × 5 | 75 × 8 | 75 × 4 | 1350 |
| **Total** | 110 | 465 | 744 | 372 | 1691 |

**Supplemental Figures and Figure Legends**

**Figure S1** **Study flowchart**


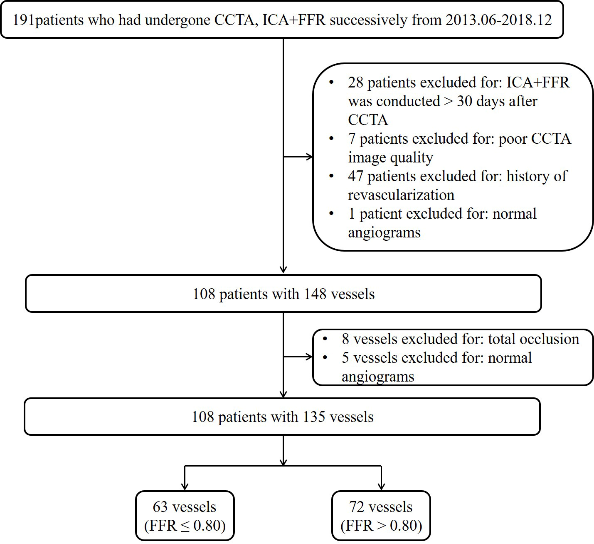


**Figure S2 Correlation and Bland-Altman plots of vessel-based and lesion-based PCAT CT attenuation in different segments of coronary artery.** Pearson correlation analyses show the correlation vessel-based and lesion-based PCAT CT attenuation in the proximal (A), middle (B) and distal segments (C), respectively. Bland-Altman plots show PCAT CT attenuation between vessel-based and lesion-based in the proximal (D), middle (E) and distal segments (F). The red dotted lines represent the mean difference (bias), and the blue dotted lines represent the 95% limits of agreement. PCAT, pericoronary adipose tissue


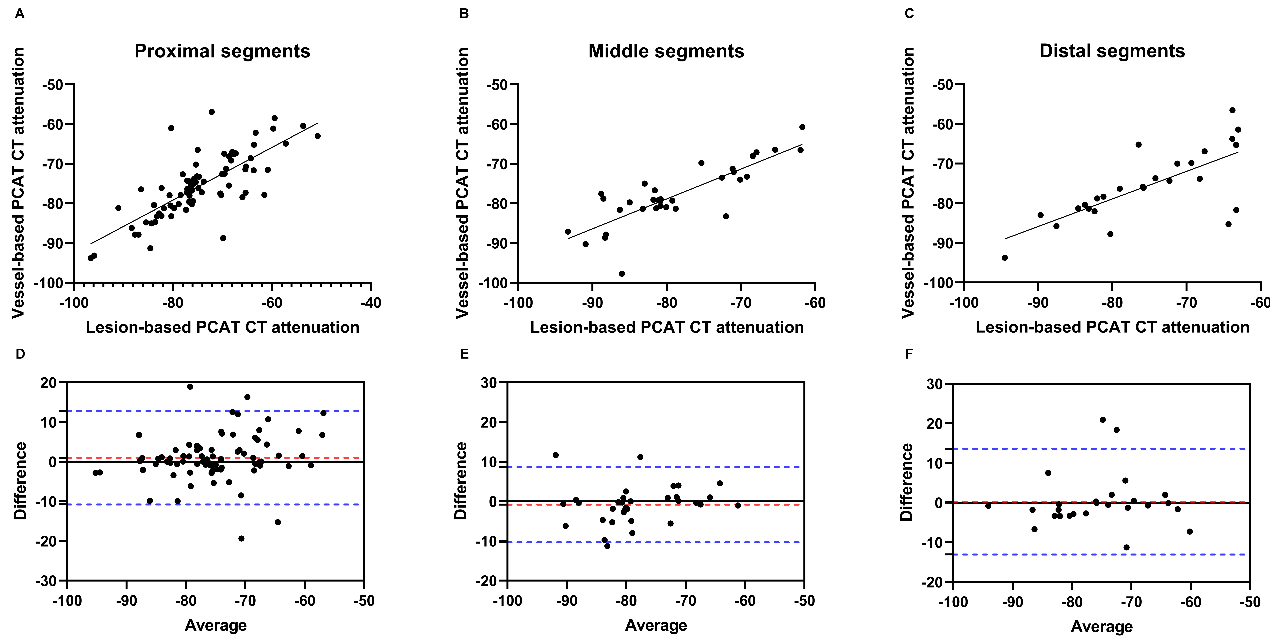

Supplement: Supplementary file 1 [file Data_Sheet_1.docx]
